# The Oral Bacterial Microbiome of Interdental Surfaces in Adolescents According to Carious Risk

**DOI:** 10.3390/microorganisms7090319

**Published:** 2019-09-05

**Authors:** Camille Inquimbert, Denis Bourgeois, Manuel Bravo, Stéphane Viennot, Paul Tramini, Juan Carlos Llodra, Nicolas Molinari, Claude Dussart, Nicolas Giraudeau, Florence Carrouel

**Affiliations:** 1 Laboratory “Systemic Health Care”, EA4129, University Lyon 1, University of Lyon, 69008 Lyon, France (C.I.) (D.B.) (S.V.) (C.D.); 2 Department of Public Health, Faculty of Dental Medicine, University of Montpellier, 34090 Montpellier, France (P.T.) (N.G.); 3 Department of Preventive and Community Dentistry, Faculty of Odontology, University of Granada, 18010 Granada, Spain (M.B.) (J.C.L.); 4 Service DIM, CHU de Montpellier, UMR 5149 IMAG, University of Montpellier, 34090 Montpellier, France

**Keywords:** oral microbiome, adolescents, carious risk, interdental microbiota

## Abstract

Adolescence is closely associated with a high risk of caries. The identification of specific bacteria in an oral microniche, the interdental space of the molars, according to carious risk can facilitate the prediction of future caries and the anticipation of the progression or stabilization of caries in adolescents. A cross-sectional clinical study according to the bacteriological criteria of interdental healthy adolescents and carious risk factors—low and high—using a real-time polymerase chain reaction technique was conducted. The presence of 26 oral pathogens from the interdental microbiota of 50 adolescents aged 15 to 17 years were qualitatively and quantitatively analyzed. Bacteria known to be cariogenic (*Bifidobacterium dentium*, *Lactobacillus* spp., *Rothia dentocariosa*, *Streptococcus cristatus*, *Streptococcus mutans*, *Streptococcus salivarius*, *Streptococcus sobrinus*, and *Streptococcus wiggsiae*) did not present differences in abundance according to carious risk. Periodontal bacteria from the red complex are positively correlated with carious risk. However, only 3 bacteria—*S. sobrinus*, *E corrodens* and *T. forsythia*—presented a significant increase in the highest group. Estimating the risk of caries associated with bacterial factors in interdental sites of molars in adolescents contributes to the better definition of carious risk status, periodicity and intensity of diagnostic, prevention and restorative services.

## 1. Introduction

In 2017, the three most common causes of the global burden of diseases in the world were oral disorders (3.47 billion, 95% UI 3.27–3.68), headache disorders (3.07 billion, 95% UI 2.90–3.27), and tuberculosis, including latent tuberculosis infection (1.93 billion, 95% UI 1.71–2.20) [1]. The same year, the prevalence of caries on permanent teeth was 23,019,992,000 [1]. Dental caries associated with periodontal disease are, together, the most prevalent microbe-mediated human disease worldwide [2].

Dental caries is a biofilm-mediated, sugar-driven, multifactorial, dynamic disease that results in the phasic demineralization and remineralization of dental tissues [3]. Dental caries is the result of dissolution of the tooth mineral by a reduction in pH due to the sustained fermentation of carbohydrate by bacteria in a local biofilm structure that limits the ability of saliva to wash away or buffer the acidic metabolic products [4,5,6]. The biofilm diversity of tooth surfaces is influenced by carbohydrate consumption and a surface’s health status [7]. Caries are associated with dysbiosis of the tooth-colonizing microbiota, characterized by the accumulation of aciduric and acidophilic bacteria [8]. The balance between pathological and protective factors influences the initiation and progression of caries [3].

The risk for developing caries in individuals depends on factors such as the immune system and oral microbiome, which themselves are affected by environmental and genetic determinants [9]. Interdental space is one of the main sites at risk of dental caries. Indeed, due to their anatomical arrangement and location, the biological interdental space in healthy subjects with an estimated diameter of 0.6–1.1 mm is an ecological microniche protected externally by the papillary gingiva [4,10]. Interdental spaces facilitate the structuration and accumulation of oral biofilms [11]. The saliva, due to anatomic accessibility constraints, cannot circulate favorably. Thus, without adequate saliva, the oral clearance of sugary or acidic foods will be longer, and less urea is available to help raise the plaque biofilm pH [12]. Moreover, accessibility to interdental spaces by conventional methods of individual prophylaxis is restricted. If toothbrushing is optimal for cleaning occlusal, facial and lingual/palatal surfaces of teeth, none of the toothbrushing methods is efficient for eliminating the interproximal supragingival dental plaque or disrupting the biofilm [13]. The effectiveness of plaque removal after brushing has been estimated at approximately 42% [14].

Adolescence is closely associated with a high risk and progression for caries. In total, 67% of US teens have experienced tooth caries, with untreated decay in 20% [15]. The interproximal faces of the molars are mainly affected [16]. The prevalence of caries has been reported to be 39% at the age of 12, increasing to 72% at the age of 20–21 [17]. Enamel lesions on the proximal surfaces among 16-year-olds account for more than 80% of all caries lesions on these surfaces, no matter whether the caries prevalence in the population is high or low [18]. During adolescence, the changes in dietary habits associated with excessive high carbohydrate consumption, sugary sodas or popular energy drinks, foods and snacking could contribute to alter the balance of the oral microbiome [19,20]. Admittedly, demineralization can be inhibited by salivary components, antibacterial agents, and fluoride or reversed by remineralization, which requires calcium, phosphate, and fluoride [21]. However, in interdental spaces, the conditions conferred by the topical application of fluoride, a priority in childhood and adolescence for the remineralization of enamel and brought daily by toothpaste, then conveyed by saliva, are not met to provide optimal protection. Adolescents′ compliance associated with poor oral hygiene is advanced [22]. Oral hygiene and toothbrushing are not always a priority [23]. Access to community prevention interventions for promoting adolescent oral health, i.e., policy, educational activities, supervised toothbrushing programs, is more complicated because these mainly focus on early childhood and childhood [24].

Previous studies have focused on the oral microbiota of children with and without tooth decay. Moreover, most oral microbiology studies are based on pooled samples [25], rather than characterizing potential differences in microbial composition between discrete sites on teeth. Ribeiro and colleagues have recently studied bacterial diversity in occlusal biofilms and its relationship with the clinical surface diagnosis and dietary habits of 12-year-old children [6]. Our research is the first to target an oral microniche, which represents the interdental (ID) space, using real-time polymerase chain reaction (PCR) in order to analyze qualitatively and quantitatively the bacterial composition and to study its relation with caries risk factors in adolescents.

Better information on changes in community structure—taxonomic identity and abundance—that evolve in the aggressive ecosystem of a potentially cariogenic biofilm is thus significant for caries risk assessment and for progress in developing preventive strategies [6]. Caries-risk assessment models currently involve a combination of factors including diet, fluoride exposure, a susceptible host, and microflora that interplay with a variety of social, cultural, and behavioral factors [26]. Our hypothesis is that the identification of specific bacteria according to the degree of severity of carious risk can contribute to risk assessment to predict future caries and anticipate caries progression or stabilization in the adolescent.

This study investigated the changes in biofilm composition of interdental surfaces in posterior permanent teeth according to carious risk. Data to identify bacteria from the microbiome associated with caries risk will be emphasized.

The hypotheses are: (i) The quantification of the oral microbiome at the interproximal surface of the tooth could contribute to the assessment of carious risk and personalized clinical decision-making, (ii) the qualitative differentiated analysis of the microbiome could help to predict interproximal adolescence caries development, (iii) the identification of specific bacterial species could help to develop novel approaches in their diagnosis and management of carious lesions.

## 2. Materials and Methods

**Figure 1:**
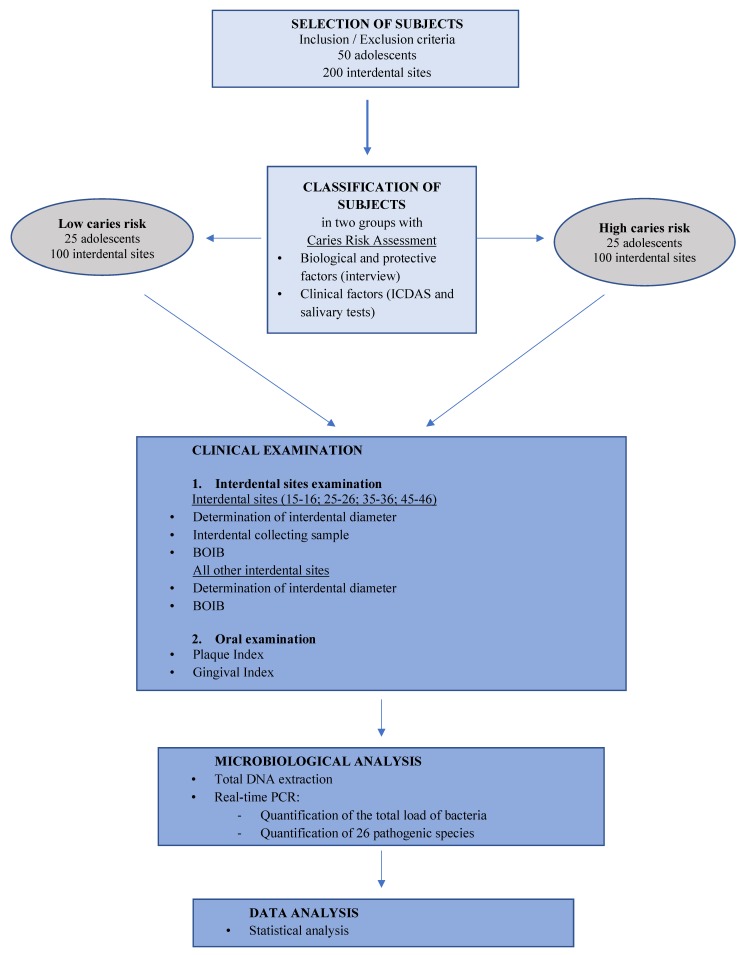
Workflow of the experiment. BOIB: Bleeding on Interdental Brushing; DNA: Deoxyribonucleic acid; ICDAS: International Caries Detection and Assessment System; PCR: Polymerase Chain Reaction.

The Microbiota of Interdental Space of Adolescent according to Risk of Caries (MIARC) trial is a cross-sectional observational clinical study. The workflow of the experiment is described in Figure 1.

### 2.1. Study Population

Fifty subjects (male and female) were recruited between November and December 2018 from a pool of first-time volunteers who were referred to the Department of Public Health of the Oral Medicine Hospital of Montpellier, France. Written informed consent was obtained from all enrolled subjects in accordance with the Declaration of Helsinki and authorization of the ethics council. The 50 adolescents were selected based on inclusion criteria and carious risk classification to have 25 subjects in the high caries risk (HCR) group and 25 subjects in the low caries risk (LCR) group.

The inclusion criteria were: (i) Age 15–17 years old, (ii) presence of teeth (15, 16, 25, 26, 35, 36, 45, and 46), (iii) accessibility of the interdental space for the four sites (15–16, 25–26, 35–36, and 45–46) by the interdental brush in each subject, (iv) the presence of at least 22 natural teeth, (v) good understanding of the French language, (vi) one of the parents accepts the terms of the study and signs the written informed consent, (vii) adolescent accepts the terms of the study and signs the written informed consent.

The clinical inclusion criteria for each premolar-molar interdental site were: (i) Accessibility of the interdental site for the four sites (15–16, 25–26, 35–36, and 45–46) by the interdental brush in each subject, (ii) no interdental caries or prosthetics restorations, (iii) no interdental diastema, (iv) no clinical sign of inflammation such as redness, swelling, or bleeding on probing (BOP) after 30 s, (v) no pocket depth (PD) > 3 mm or clinical attachment loss (CAL) > 3 mm, and (vi) the subjects were judged to be free of gingivitis or periodontitis.

The exclusion criteria were: (i) Smokers, (ii) subjects with any other concomitant systemic disease, (iii) subjects with daily medication, (iv) subjects with an orthodontic appliance, (v) subjects who have taken antibiotics in the past three months, (vi) subjects regularly using interdental brushes and/or dental floss and/or mouthwash, and (vii) subjects unable to answer questions and non-cooperative.

### 2.2. Ethical Approval and Informed Consent

This study was carried out in accordance with the ethical committee of Sud-Est VI Clermont-Ferrand (Approval number: AU1371) and the National Commission of Informatics and Liberties, France (2116544 v 0). The National Agency for the Safety of Medicines and Health Products (ANSM) approved it on February 13, 2017 (ID-RCB ref: 2017-A00425-48). This study was registered with ClinicalTrials.gov (identification number ID: NCT03700840).

### 2.3. Classification of Subjects According to Carious Risk

One trained and calibrated dentist, experienced in the clinical indices, realized the clinical examination.

Subjects were classified into two carious risk groups: Low and high risk. In this study, the classification of subjects according to their carious risk was adapted from the caries-risk assessment of the American Academy of Pediatric Dentistry that is based on biological, protective and clinical factors [27].

First, to know the biological and protective factors, the dentist interviewed the patient face-to-face. Questions referred to socioeconomic status (mother′s and father’s occupation based on French socio-economic classifications), snacks, brushing, regular visits and dental care (Table 1).

Then, a clinical examination was performed to fulfil the questions concerning the clinical factors (Table 1). The participants were asked to refrain from oral hygiene measures, eating and drinking for two hours before clinical examination and interdental sampling.

The presence of active caries and interproximal lesions were measured by the International Caries Detection and Assessment System (ICDAS). This clinical scoring system allows the detection and the assessment of caries activity. ICDAS was developed for use in clinical research, clinical practice and for epidemiological purposes. This scoring system can be used on coronal surfaces and root surfaces and can be applied to enamel caries, dentine caries, non-cavitated lesions (contrary to many systems) and cavitated lesions. The ICDAS II system has two-digit coding for the detection criteria of primary coronal caries. The first one is related to the restoration of teeth and has a coding that ranges from 0 to 9. The second digit ranges from 0 to 6 and is used for coding the caries. A compressed air syringe was used to dry the teeth during the ICDAS assessment [28].

The salivary tests were performed for all subjects using Saliva-Check BUFFER (GC, Sucy-en-Brie, France). The tests aimed to investigate hydration, salivary consistency, resting saliva pH, stimulated saliva flow, stimulated saliva pH and saliva buffering capacity. All tests were performed according to the instructions of the manufacturer to detect low, moderate, or high salivary risk.

The subject was classified as “high carious risk” if at least one “yes” was selected in the column “high risk”. The subject was classified as “low carious risk” only if no “yes” was selected in the column “high risk” for the biological and clinical findings, and “yes” was selected for the protective factors.

### 2.4. Clinical Examination and Interdental Sample Collection

For all subjects, the same four interdental sites (15–16, 25–26, 35–36, and 45–46) were assessed (total 200 sites). The interdental diameter was determined using a dedicated probe—the CURAPROX IAP calibration probe (Curaden, Kriens, Switzerland). This probe is a graduated conical instrument with a rounded end. The working portion includes colored bands from the tip to the base corresponding to interdental brushes (IDB) by increasing diameter. The largest section of each colored band corresponds to the cleaning efficiency diameter of the respective brush. This made it possible to select the calibrated IDB (Curaden) appropriate to the diameter of the interdental space. Each previously selected tooth was isolated using sterile cotton rolls, and the interdental biofilm as removed using this sterile IDB. For each sample, the IDBs were placed in 1.5-mL sterile microcentrifuge tubes and stored at 4 °C for further laboratory treatment.

The Bleeding on Interdental Brushing Index (BOIB) [29] was evaluated on the four interdental sites (15–16, 25–26, 35–36, and 45–46), as was the bleeding response to the horizontal pressure applied in the interdental area by a calibrated IDB. After 30 s, bleeding at each gingival unit was recorded according to the following scale: 0, absence of bleeding after 30 s; and 1, bleeding after 30 s. Then, interdental diameters and the BOIB were evaluated for all other interdental sites.

Clinical measurements including first the Gingival Index (GI) and, second, the Plaque Index (PI), were performed on the 4 sides (buccal, lingual/palatal, mesial, distal) of 6 teeth (12, 16, 24, 36, 32, 44) (Silness–Löe Index) [30]. To evaluate the GI, the tissues surrounding each tooth was divided into 4 gingival scoring units: Distal facial papilla, facial margin, mesial facial papilla and lingua gingival margin. A periodontal probe was used to assess the bleeding potential of the tissues with a score from 0–3 (0: Absence of inflammation to 3: Severe inflammation). The scores of the four areas of the tooth were summed and divided by four to obtain the GI for the tooth. The index for the patient was calculated by summing the indices for all six teeth and dividing by six. Then, the determination of the PI recorded both soft debris and mineralized deposits on the 6 teeth. Each of the four surfaces of the teeth gave a score from 0–3. The scores from the four areas of the tooth were added and divided by four in order to give the plaque index (0: no plaque to 3: abundance of soft matter within the gingival pocket and/or on the tooth and gingival margin). The index for the patient was obtained by summing the indices for all six teeth and dividing by six.

### 2.5. Microbiological Analysis

#### 2.5.1. Total Deoxyribonucleic Acid (DNA) Extraction

Total DNA was isolated from the interdental brushes using the QIAcube HT Plasticware and Cador Pathogen 96 QIAcube HT Kit (Qiagen, Hilden, Germany), according to the manufacturer’s guidelines. The elution volume used in this study was 150 μL. DNA quality and quantities were measured using an ultraviolet spectrophotometer at 260 and 280 nm. The DNA sample was considered pure if the A260/A280 ratio was in the range of 1.8–2 and the A260/A280 ratio was in the range of 2–2.2.

#### 2.5.2. Quantitative Real-Time PCR Assays

Quantitative real-time PCR was carried out for Total Bacterial Count (TB) and for 26 pathogens: *Aggregatibacter actinomycetemcomitans* (*Aa*), *Porphyromonas gingivalis* (*Pg*), *Tannerella forsythia* (*Tf*), *Treponema denticola* (*Td*), *Prevotella intermedia* (*Pi*), *Parvimonas micra* (*Pm*), *Fusobacterium nucleatum* (*Fn*), *Campylobacter rectus* (*Cr*), *Eikenella corrodens* (*Ec*), *Prevotella nigrescens* (*Pn*), *Campylobacter gracilis* (*Cg*), *Capnocytophaga ochracea* (*Co*), *Actinomyces odontolyticus* (*Ao*), *Veillonella parvula* (*Vp*), *Streptococcus mutans* (*Smutans*), *Streptococcus mitis* (*Smitis*), *Streptococcus sobrinus* (*Ssob*), *Streptococcus salivarius* (*Ssal*), *Streptococcus sanguinis* (*Ssan*), *Streptococcus cristatus* (*Scri*), *Rothia dentocariosa* (*Rd*), *Bifidobacterium dentium* (*Bd*), *Scardovia wiggsiae* (*Sw*), *Clostridium cluster IV* (*ClosIV*) (*Clostridium leptum* subgroup, includes *Faecalibacterium* (*Fusobacterium*) *prausnutzii*), *Clostridium cluster XIVa* and *XIVb* (*ClosXIV*) (*Clostridium coccoides*–*Eubacterium rectale* subgroup), and *Lactobacillus* spp. (*Lspp*).

Simplex quantitative real-time PCR assays were performed in a volume of 10 µL composed of 1× SYBR Premix Ex Taq™ (Tli RNaseH Plus) (TaKaRa, Shiga, Japan), 2 μL of DNA extract and each primer at 1 µM. The bacterial species-specific PCR primers used in this study were provided by Institut Clinident SAS (Aix-en-Provence, France) and manufactured by Metabion international AG (Planegg, Germany). The bacterial primers used in this study are derived from sequences already published and have been adapted to the real-time PCR conditions (Supplementary Table S1).

The assays were performed on the Rotor-Gene Q thermal cycling system (Qiagen, Hilden, Germany) with the following program: 95 °C for 30 s, followed by 40 cycles of 10 s at 95 °C, 10 s at the appropriate annealing temperature (Supplementary Table S1), and 35 s at 72 °C. For the total bacterial load and that of all species, a final melting curve analysis (70–95 °C in 1 °C steps at 5 s increments) was performed. Fluorescence signals were measured every cycle at the end of the extension step and continuously during the melting curve analysis. The resulting data were analyzed using Rotor-Gene Q Series software (Qiagen, Hilden, Germany).

Serial dilutions of a bacterial standard DNA provided by Institut Clinident SAS (Aix-en-Provence, France) were used in each reaction as external standards for the absolute quantification of the targeted bacterial pathogens. The standard bacterial strains used for standard DNA production came from DSMZ (Germany), CIP Collection of Institut Pasteur or from BCMM/LMG Bacteria Collection: *Aa* (DSM No. 8324), *Pg* (DSM No. 20709), *Tf* (CIP No. 105220), *Td* (DSM No. 14222), *Pi* (DSM No. 20706), *Pm* (DSM No. 20468), *Fn* (DSM No. 20482), *Cr* (LMG No. 7613), *Ec* (DSM No. 8340), *Pn* (DSM No. 13386), *Cg* (DSM No. 19528), *Co* (DSM No. 7271), *Ao* (DSM No. 43760), *Vp* (CIP No. 60.1), *Rd* (DSM No. 43762), *Bd* (DSM20436), *Sw* (DSM No. 22547), *Lspp* (CIP No. 102237), *Smitis* (DSM No. 12643), *Smutans* (DSM No. 20523), *Ssob* (DSM No. 20742), *Ssal* (DSM No. 20067), *Ssan* (DSM No. 20068), *Scri* (DSM No. 8249), *ClosIV* (DSM 753), and *ClosXIV* (DSM No. 935).

The pathogenic strains were cultivated on the appropriate selective media. The total number of cells (number of colony-forming units) was enumerated three times using a Neubauer chamber. Serial dilutions ranging from 10xE+2 to 10xE+12 cells were utilized, and each of these dilutions was enumerated in duplicate. The DNA from each of these dilutions was extracted. A standard curve for each pathogen was generated as a plot between the crossing point (cycle number) and the initial cell count. The absolute counts of pathogen were determined using these calibration curves [31].

The limit of quantification (LOQ) of the method is summarized in Supplementary Table S1.

### 2.6. Statistical Analysis

#### 2.6.1. Sample Size

With an alpha error of 5% (2-sided test), a power of 80%, an intraclass correlation coefficient of 0.8, and a mean difference of bacteria counts between the two caries risk groups of 1,300,000, a total of 200 sites (which means 50 subjects i.e., 25 subjects per caries risk group) was necessary.

#### 2.6.2. Statistical Tests

The statistical analysis consisted of three main steps: Producing descriptive summaries of the data, modeling the data using a mixed (linear) model and assessing the correlations between bacterial abundances. Prior to these steps, we transformed the original count data to handle missing data points, namely, the measurements that fell under the quantification threshold (LOQ) of the quantitative real-time PCR device. The missing values for a given species were replaced by half of the corresponding quantification thresholds given in Supplementary Table S1. We performed simulations to ensure that this simple strategy provided a reasonable estimation of the mean and standard deviation of the original count distribution. To test for potential effects of gender, interdental space, and the location of each site, we used a mixed linear model for the log-count abundance of each species at a measured site. This model includes three categorical variables as fixed effects (gender, mouth location, and interdental space) and one categorical variable as a random effect (subject). This random effect was introduced for a subject to model the correlation between the four sites of a given subject. Each coefficient in the regression was tested against the null hypothesis, which indicates that the coefficient is zero using a likelihood ratio test, and we reported that *p*-values less than 0.05, 0.01, and 0.001 were low, medium, and strong evidence against the null hypothesis, respectively. To perform the correlation analysis, we used the residuals of the model described above to avoid over-estimating the inter-site correlation (sites from the same patient are positively correlated, and we observed that fixed effects can also induce a correlation among sites). The trees associated with the correlation plot were obtained by hierarchical clustering with complete linkage. The difference between the two groups of caries risk relative to age, gender, mouth location, interdental space, BOIB, PI and GI were tested with chi-square tests. Kruskal–Wallis tests were performed to compare the mean counts for the different bacterial species relative to each clinical characteristic.

All statistical analyzes and associated plots were performed using the R environment (R Core Team, 2015), specifically the lme4 package [32], to estimate the mixed model.

## 3. Results

### 3.1. Age, Gender, and Clinical Characteristics in the Two Carious Risk Groups

Table 2 summarizes the age, the sex, and clinical assessments of the study groups. The two groups were each composed of 25 subjects. The age, gender and clinical characteristics were similar in the two groups (Mann–Whitney and chi-square tests). The low caries risk (LCR) group was composed of 18 females and 7 males, whereas the high caries risk (HCR) group was composed of 15 females and 10 males. The mean age was 16.98 ± 0.77 years. The mean number of teeth present was 27.6 ± 1.1 (excluding wisdom teeth). Missing teeth (1.3%) were due to orthodontic treatment or agenesis (0.95%) and caries (0.35%). The mean BOIB score in the low risk group was 96.52% and 96.55% in the high-risk group. No problems due to oral hygiene were observed (fair plaque index 1.0–1.9, gingival index 1.1–2.0). The interdental spaces studied had a diameter between 0.8 and 1.1 mm.

### 3.2. Quantification of the Total Genome Count and Bacteria Count According to Carious Risk

**Figure 2:**
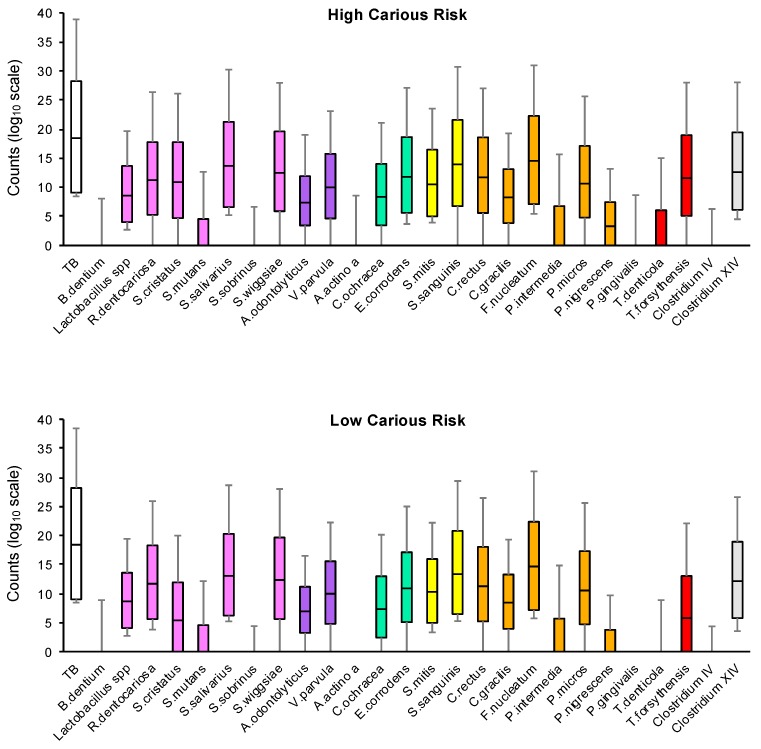
Abundance of bacterial species among the interdental sites in the low carious risk and high carious risk groups. The counts are reported on a log10 scale. Each box represents the first quartile, median quartile, and third quartile, from bottom to top. The first box on the left (TB) corresponds to the total bacteria. The colors in boxes refer to (i) the colors of the Socransky complexes for the purple, green, yellow, orange, and red colors, (ii) cariogenic bacteria for the pink color and (iii) bacteria from the *clostridium* group for the gray color. TB, total bacterial load.

The mean counts for the total bacterial load and that of the 26 evaluated species in the interdental biofilm according to carious risk are reported in Figure 2 and Table 3. An average of approximately 10^9.4^ bacteria was collected in one interdental space for both groups. The quantity of total bacteria and of bacteria tested was not significantly modified except for *T. forsythia*, *E. corrodens*, and *S. sobrinus*, which were significantly increased in the HCR group relative to the LCR group. *T. forsythia* was 26.3 times, *E. corrodens* 4.7 times and *S. sobrinus* 3.3 times higher in the HCR group than in the LCR group.

In the HCR group, all species tested were detected. The most abundant species were *F. nucleatum* (10^7.4^ bacteria in one ID space), *S. salivarius* (10^7.05^ bacteria in one ID space) and *S. sanguinis* (10^7.05^ bacteria in one ID space). The least abundant species were *Clostridium IV* (10^0.52^ bacteria in one ID space), *A. actinomycetencomitans* (10^0.55^ bacteria in one ID space), and *S. sobrinus* (10^0.56^ bacteria in one ID space).

In the LCR group, the most abundant species were *F. nucleatum* (10^7.43^ bacteria in one ID space), *S. sanguinis* (10^6.94^ bacteria in one ID space) and *S. salivarius* (10^6.75^ bacteria in one ID space). *A. actinomycetencomitans* and *P. gingivalis* were not detected.

Table 4 describes the distribution of pathogens according to sites and subjects. *Lactobacillus spp*., *S. salivarius*, *S. mitis*, *F. nucleatum*, and *Clostridium XIV* were detected in all subjects and in all interdental spaces, whatever the carious risk. Other bacteria were not expressed in each interdental site nor in each subject. For example, *P. gingivalis* was detected in 11% of HCR subjects and 12% of interdental sites from HCR subjects, but this bacterium was not detected in LCR subjects. *E. corrodens* was observed in all subjects, in 100% of interdental sites from HCR subjects and in 96% of interdental sites from LCR subjects.

### 3.3. Effects of Gender, Interdental Diameter and BOIB on the Total Genome Count and Bacteria Count

Table 5 summarizes the interactions between caries risk and gender, IDB size or BOIB. For the majority of the pathogens tested, the gender, the interdental diameter and the BOIB had no significant effect on the bacteria count. However, the sex significantly impacted the quantity of *E. corrodens*, *P. nigrescens* and *S. mutans*. The IDB size significantly modified the bacteria counts of *T. denticola*, *P. nigrescens*, *C. orchracea*, *V. parvula*, *B. dentium*, *S. cristatus*, *Clostridium IV*, and *Clostridium XIV*. The BOIB significantly influenced the quantity of *T. denticola*, *C. rectus*, *E. corrodens*, *S. salivarius*, and *S. sanguinis*.

**Figure 3:**
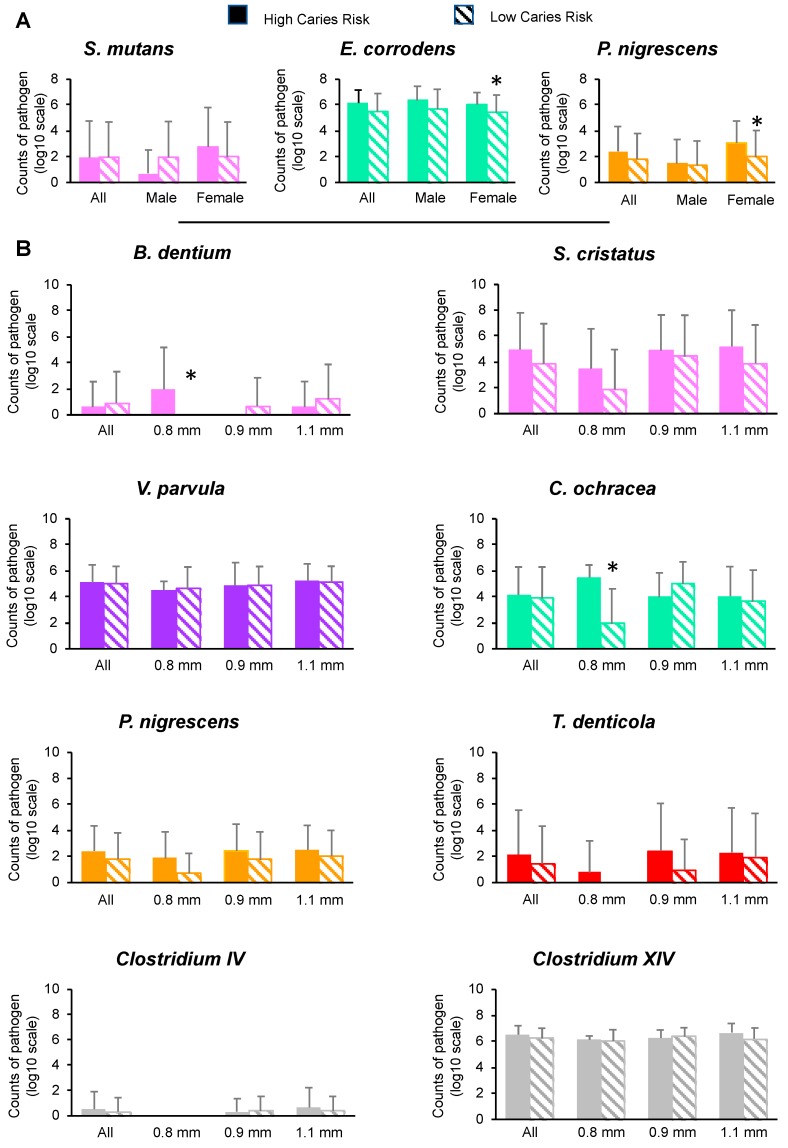
Abundance of bacterial species according to sex and interdental diameter in the low carious risk group and in the high carious risk group. The counts are reported on a log10 scale. Total counts from each pathogen were averaged across sites in each subgroup. Error bars represent standard deviations. This stratified analysis is restricted to those situations where the interaction between caries risk and sex (**A**) or caries risk and IDB size (**B**) on bacterial counts is significant (*p* < 0.05) (detailed results not shown). Comparisons: * *p* < 0.05, by using SUDAAN 7.0 (procedures DESCRIPT and REGRESS) to account for clustering (multiple sites within the subjects). The colors in boxes refer to (i) the colors of the Socransky complexes for the purple, green, yellow, orange, and red colors, (ii) cariogenic bacteria for the pink color and (iii) bacteria from the *clostridium* group for the gray color. TB: total bacterial load.

The counts of *E. corrodens* and *P. nigrescens* were lower in the LCR group than in the HCR group for males and females, but the decreases were significant only for females (Figure 3). No significant effect was observed for *S. mutans* for males and females. A significant decrease in the counts of *B. dentium,* and *C. ochracea* was observed for IBD of 0.8 mm. For other bacteria tested and other diameters of IDB, no significant effect was observed.

Table 6 details the effect of BOIB on bacterial count according to caries risk. The quantity of *S. salivarius* and *S. sanguinis* significantly increased in the HCR group, whereas no significant effect was observed for the LCR group. *E. corrodens* was not significantly correlated with the BOIB. *T. denticola* was significantly increased with the BOIB in the LCR group but not in the HCR group. *C. rectus* was correlated with the increase in BOIB in both groups.

### 3.4. Pathogen Correlations According to Carious Risk

**Figure 4:**
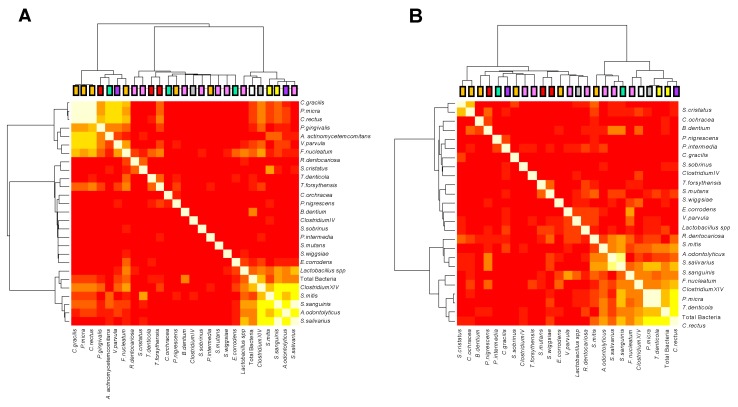
Correlation plot of the abundances of the bacterial species, corrected for age, interdental space and individual-specific effects. (**A**) High caries risk group, (**B**) low caries risk group. Yellow indicates positive correlations, whereas red indicates the absence of correlations. The colored leaves on the top dendrogram represent (i) the colors of the Socransky complexes for the purple, green, yellow, orange, and red colors, (ii) cariogenic bacteria for the pink color and (iii) bacteria from the *clostridium* group for the gray color.

The dendrogram (Figure 4) underscores the correlations between the 26 pathogenic species and the ID sites for the HCR group and the LCR group. Even after the removal of the fixed effects related to interdental space and age, and the subtraction of the inter-site correlations, the matrix still revealed a strong correlation structure, which appeared as two groups (or clusters) of correlated species for the HCR group and one for the LCR group.

The first cluster of the HCR group was composed of *C. gracilis*, *P. micra*, *C. rectus*, *P. gingivalis*, *A. actinomycetemcomitans*, *V. parvula*, and *F. nucleatum*, whereas the second cluster was composed of *Lactobacillus* spp., *Clostridium XIV*, *S. mitis*, *S. sanguinis*, *A. odontolyticus*, and *S. salivarius*.

The cluster from the LCR group was composed of *S. mitis*, *A. odontolyticus*, *S. salivarius*, *S. sanguinis*, *F. nucleatum*, *Clostridium XIV*, *P. micra*, *T. denticola*, and *C. rectus*.

## 4. Discussion

This study provides a comprehensive survey of the interdental microbiota in adolescents, a target group that remains poorly explored, according to carious risk. To our knowledge, there is no scientific reference in the literature that jointly targets a cross-sectional clinical study (MIARC), according to the bacteriological criteria of interdental healthy adolescents, caries risk factors and the use of a real-time PCR technique. A few studies focused on the carious microbiota of adolescents, but these studies were concerned with the subgingival microbiota without specific location, during and/or after orthodontic treatment [33,34], and quantified few bacteria (*A. actinomycetemcomitans*, *P. gingivalis*, *P. intermedia*, *T. forsythia*) [35].

Most research investigating the commensal oral microbiome are focused on disease or are restricted in methodology. To diagnose and treat caries at an early and reversible stage, an in-depth definition of health is indispensable [36]. Our research option consisted of carrying out real-time PCR analysis of the interdental microbiota of caries-free sites in healthy adolescents with or without a carious risk. All pathogens considered in our study have been before identified in oral samples of children, adolescents, or young adults [37,38,39,40]. Our study did not only focus on cariogenic bacteria because the classification of oral bacteria seems more complex. Effectively, some studies demonstrated a positive association between periodontitis and caries, whereas others demonstrated a negative association [41,42,43]. These studies observed clinical signs, and only one study considered the microbiota, but the subjects were clinically affected by these oral diseases [44]. Qualitatively and quantitatively, the presence of 26 oral pathogens was analyzed. Among them were bacteria commonly classified as cariogenic bacteria—*B. dentium*, *Lactobacillus* spp., *R. dentocariosa*, *S. cristatus*, *S. mutans*, *S. salivarius*, *S. sobrinus*, *S. wiggsiae*; bacteria considered periodontopathogenic—bacteria from the purple complex (*A. odontolyticus*, *V. parvula*), the green complex (*A. actinomycetemcomitan*, *C. orchrocea*, *E. corrodens*), the yellow complex (*S. mitis*, *S. sanguinis*), the orange complex (*C. rectus*, *C. gracilis*, *F. nucleatum*, *P. intermedia*, *P. micra*, *P. nigrescens*), the red complex (*P. gingivalis*, *T. denticola*, *T. forsythia*); and others such as *Clostridium IV* and *Clostridium XIV*.

A particular focus of our study is a Caries Risk Assessment (CRA) system. Indeed, the carious lesion is a multifactorial disease principally due to carious biofilm and sugar consumption. Caries-risk assessment models currently involve a combination of factors including diet, fluoride exposure, a susceptible host, and microflora that interplay with a variety of social, cultural, and behavioral factors [26]. The initiation of dental caries results from the balance between risk and protective factors. This interplay between factors underpins the classification of individuals and groups into caries risk categories, allowing an increasingly tailored approach to care. The classification criteria for CRA systems were determined by combining scientific evidence and expert opinion. From these CRAs, practitioners analyze the various clinical and social factors of a patient and can thus assign a carious risk status [45]. For low-risk patients, there is no necessity for further preventive professional treatment, and they should be offered an extended follow-up [46]. For high-risk patients, preventive actions must be taken to reduce the incidence and severity of future carious lesions [46]. This individual scheduling of preventive and follow-up activity better appropriates the use of dental resources and lowers dental costs for certain individuals [46]. At present, the criteria needed to perform quantitative caries risk assessment evaluations are still incomplete [26].

Few CRA classifications consider the quantification of bacteria, and generally such bacteria are singular and unique. In these few CRA classifications, the quantification of *S. mutans* in the saliva has been considered [47]. However, the results concerning the link between *S. mutans* and the development of dental caries is not clear. Some studies demonstrated a real association between *S. mutans* and the carious lesion, whereas others revealed no clear association [48]. Moreover, the amount of *S. mutans* needed to initiate the carious lesion varies according to the study [49]. Thus, to use this bacterium or other bacteria as a biomarker in a CRA classification, it is necessary to identify specific biomarkers associated with carious lesions and to normalize their quantification.

Concerning bacteria classified as cariogenic bacteria (*B. dentium*, *Lactobacillus* spp., *R. dentocariosa*, *S. cristatus*, *S. mutans*, *S. salivarius*, *S. sobrinus*, *S. wiggsiae*), no significant difference was observed in the HCR subjects relative to the LCR subjects. Therefore, these bacteria do not represent good predictive factors for CRA. Cariogenic bacteria are acidogenic and acid-tolerant species [50].

For many years, *S. mutans* was considered the major oral pathogen implicated in the initiation of caries. However, this role has been questioned [51]. Effectively, caries have been observed without the presence of *S. mutans* [50,52,53,54]. In our study, *S. mutans* was expressed in small quantities (10^1.97^ for the LCR and 10^1.95^ for the HCR group). Our results confirmed that *S. mutans* alone could not be used to predict carious risk as previously demonstrated by Gross and colleagues [50]. A CRA classification that uses the quantification of *S. mutans* in the saliva must be used with care [21]. In some studies, caries was associated with the absence of *S. mutans* and the presence of *Lactobacillus* [55,56], *B. dentium* [55], and *S. wiggsiae* [57].

*S. sobrinus*, closely related to *S. mutans*, is described as a major bacterium in the apparition of carious lesions [58]. This pathogen was significantly increased by three times in the HCR group relative to the LCR group, and it could also be a predictive marker for caries, as described by Gross and colleagues for the supragingival biofilm [50]. However, contrary to this previous study, this pathogen was expressed only in 20% of the subjects from the HCR group, which limits its detection and its potential to be an interesting predictive marker of caries.

*S. salivarius* has been associated with caries due to its high cariogenic capacity [55]. Moreover, in a rat model, this pathogen was able to induce caries. Contrary to these results, in our study, this pathogen was expressed in all subjects at equal levels in both groups [59]. These divergent results could be explained by the fact that some *S. salivarius* strains such as *S. salivarius* M18 have probiotic activity and help to fight against cariogenic bacteria [60].

*S. wiggsiae* is classified as cariogenic because it is able to tolerate acid and to produce acid from several sugars at low pH [65]. This pathogen was significantly associated with severe-early childhood caries and was also observed in adolescents presenting initial carious lesions with fixed orthodontic appliances [61,62]. Contrary to these studies, our results indicated that *S. wiggsiae* was highly expressed whatever the carious risk and in more than 84% of subjects in both groups.

*S. cristatus* is considered an important cariogenic species due to its association with childhood caries [57,63]. Our results are in accordance with those of Dzidic and colleagues because, the quantity of *S. cristatus* is 13 times higher in HCR subjects but not significantly [63]. This pathogen is known to be inversely correlated to the presence of *P. gingivalis* [64]. Our results are in accordance with this because *P. gingivalis* represented 10^0.35^ counts in one ID space, whereas *S. cristatus* represented 10^4.42^ counts in one ID space.

The relationship between *Lactobacillus* and caries is well established [65,66]. *Lactobacillus* spp. produces water-insoluble polysaccharides that promote bacterial attachment to the tooth surface. Therefore, other bacteria are able to fix the organic acids that are confined that modify the microenvironment to enrich the aciduric microflora [67]. Although *lactobacilli* play an important role in the prognosis of caries, they are unlikely to play an important role in the development of caries [68,69]. Therefore, the number of salivary *lactobacilli* may be indirectly related to the progression of caries. Our study revealed that *Lactobacillus* spp. was present in all caries-free subjects, but the rate was higher in the HCR group, although not significant.

*B. dentium* was isolated from the oral biofilm and from saliva [70]. *Bifidobacterium* was isolated from 80% of plaque samples from early childhood caries patients [55,71] and from the saliva of children with a higher frequency in caries-affected children than in caries-free children [72]. *B. dentium* was defined as a novel caries-associated bacterium with an acidogenic potential and a high fluoride tolerance [73]. Our results revealed that this pathogen is only in 24% of the subjects in the HCR and LCR groups, and no significant difference was observed between the two groups. Therefore, this bacterium cannot be used for CRA.

*R. dentocariosa* was identified as a cariogenic bacteria [74,75]. Jiang and colleagues observed that the quantity of this pathogen was higher in the saliva from children with caries than caries-free children. They suggested that it could be a predictive marker for CRA. However, our results indicated that *R. dentocariosa* was expressed in 96% of subjects from the HCR group and 100% of subjects from the LCR group with no significant difference in quantity between the two groups. Therefore, this pathogen alone cannot be used for CRA.

Studies agree that oral diseases are not only attributable to specific bacteria but that they result from dysbiosis of the oral microbiota. The apparition of oral disease could be due to the decrease of bacterial taxon and to the presence of key pathogens [76,77].

Concerning bacteria classified as periodontal bacteria, as previously described, bacteria from the blue, yellow, green, and purple complexes of Socransky are associated with oral health, whereas bacteria from the orange and red complexes are associated with dysbiosis of the microbiota and the apparition of gingivitis and, later, periodontitis [78]. Our study reveals that bacteria associated with Socransky complexes and thus with gingivitis and periodontitis are present in adolescents that present no signs of gingivitis. The higher quantity of bacteria from the red complex in the HCR group than the LCR group indicated a positive correlation between periodonthopathogenic bacteria and carious risk. Previously, Durand and colleagues demonstrated a positive correlation between periodontal disease severity and the decayed, missing, filled teeth surfaces index [44].

Concerning the red complex, our results indicated that *P. gingivalis* was not detected in the subjects of the LCR group and was only detected in 11% of the interdental sites in the HCR group. This could be explained because in both groups the quantity of *S. mutans* (10^1.97^ in the LCR group and 10^1.95^ in the HCR group) and *S. sanguinis* (10^6.94^ in the LCR group and 10^7.05^ in the HCR group) were high compared with quantity of *P. gingivalis* (not detected in the LCR group and 10^0.68^ in the HCR group). A previous study demonstrated that the growth of *P. gingivalis* was significantly inhibited by supernatants from *S. mutans* and *S. sanguinis* [79]. Moreover, *S. cristatus* (10^4.97^ in the HCR group) could also inhibit the growth of *P. gingivalis* because *S. cristatus* is able to inhibit the expression of virulence genes of *P. gingivalis* and consequently delay the growth of dental plaques [80].

*T. denticola* was detected in low quantities (10^2.17^ in the HCR group) and was higher in the HCR group than the LCR group. This bacterium was detected in interdental sites expressing bacteria from the red or the orange complex. This is in accordance with previous studies that demonstrated that *T. denticola* is unable to adhere on oral surfaces as it is only able to colonize to form oral biofilms when other periodontal bacteria are present. In subgingival dental biofilms, *T. denticola* is typically associated with *P. gingivalis* [81]. A chymotrypsin-like proteinase found within a high-molecular-mass complex on the cell surface of *T. denticola* mediates its adherence to other potential periodontal pathogens such as *P. gingivalis, F. nucleatum, P. intermedia* and *P. micra* [82].

*T. forsythia* was expressed in 96% of adolescents (83% of interdental sites) from the HCR group and was the only bacteria from the red complex that significantly increased in the HCR group relative to the LCR group. This bacterium could represent an interesting biomarker for the CRA system. However, as it was expressed in 93% of subjects and 83% of interdental spaces from the HCR group, some false negatives could appear. *T. forsythia* acts with *P. gingivalis* to initiate chronic periodontitis and was not described in carious lesions. In our study, *T. forsythia* was not correlated with *P. gingivalis* and so could act with other bacteria such as *S. cristatus* and *R. dentocariosa*, which are known to be implicated in caries [74,83]. Moreover, *T. forsythia* was correlated with *T. denticola*, confirming previous results demonstrating that *T. denticola* and *T. forsythia* are involved in protein–protein interactions through a leucine-rich repeat in proteins [84].

Bacteria from the orange complex do not seem to have a key role in carious risk because no significant difference was observed by comparing the HCR and the LCR groups. In fact, these bacteria have a key role in the initiation of subgingival microbiota dysbiosis [85], but no role in carious lesions was described.

*P. nigrescens* was previously detected in children caries [86], which is in accordance with our results indicating that this pathogen was detected in 76% of subjects from the HCR group. For this pathogen, sex significantly impacted carious risk. A higher quantity was detected in females than males and in the female HCR group relative to the female LCR group. Nakagawa and colleagues previously demonstrated that *P. nigrescens* was significantly increased in puberty compared with prepuberty in females but not in males. This could be associated with sex hormone modifications [87].

Contrary to the study of Gross and colleagues that demonstrated that *C. rectus* decreased as caries progressed, our study revealed no modification according to caries risk that could be explained because those authors sampled supragingival biofilm from carious lesions, whereas we analyzed interdental biofilms associated with a healthy surface [56]. However, our study revealed that *C. rectus* increased significantly in the HCR group with the BOIB, suggesting that this pathogen could also be associated with interdental inflammation. Indeed, *C. rectus* is known to be present in chronic gingivitis [88].

From the yellow complex, *S. mitis* and *S. sanguinis* were not significantly modified when the HCR and the LCR group were compared. This result aligns with those from previous studies indicating that *S. sanguinis and S. mitis* are significantly associated with dental health [89,90,91]. *S. sanguinis* is known to be a pioneer colonizer that permits the adhesion of other microorganisms and biofilm formation [50,92,93]. Our results indicated that the quantity of *S. sanguinis* is 90 times higher than that of *S. mutans*. This result is in accordance with the study of Caufield and colleagues, who demonstrated a higher level of *S. sanguinis* relative to the level of *S. mutans* in the saliva [94].

Of the green complex bacteria studied, only *E. corrodens* was significantly increased with carious risk. Moreover, this pathogen was expressed in each subject and in each interdental space from the HCR group and in 96% of subjects from the LCR group. These results are in accordance with those from Choi and colleagues, who demonstrated that this pathogen was present in the saliva from periodontally healthy subjects [95]. Previous studies have demonstrated that *E. corrodens* is important for the progression of periodontal disease in young subjects [95,96]. This bacterium participates in the early stage of biofilm formation by mediating the specific co-aggregation of bacteria from the oral cavity. It is a middle colonizer, linking early colonizers such as Streptococci and late colonizers such as *P. gingivalis* [97]. As our results indicated that this pathogen could be a key factor for the carious process and that it was phylogenetically close to the cluster (*Lactobacillus* spp., *Clostridium XIV*, *S. mitis*, *S. sanguinis*, *A. odontolyticus*, and *S. salivarius*) from the HCR group, it could participate, as previously described in periodontitis, in biofilm formation and could permit cariogenic bacteria to adhere to biofilm. In our study, females from the HCR group had a higher quantity of *E. corrodens* than females from the LCR group. This could be explained because females have higher caries prevalence than males [98,99,100,101]. Moreover, *E. corrodens* is associated with systemic diseases such as spinal, head, and neck infection or endocarditis [102,103,104,105,106]. Therefore, it could be a predictive marker of carious disease but also of systemic diseases.

Concerning the purple complex, *V. parvula* and *A. odontolyticus* were not linked with carious risk but were expressed in more than 90% of the interdental spaces tested. Groos and colleagues demonstrated that *V. parvula* increased with the carious lesion step [50], and Kanasi and colleagues identified it in carious lesions from children [107]. *V. parvula* is important in biofilm formation. It can co-aggregate with other microorganisms such as *S. mutans*. Indeed, *V. parvula* cannot fix itself on the surface of teeth and attaches to *S. mutans* [108]. *A. odontolyticus* also acts in the early formation of biofilm [109]. *A. odontolyticus* was previously detected in carious lesions [86,110,111].

A recent study demonstrated that *Clostridium* was positively and significantly correlated to caries and pigment in primary dentition [112]. However, even in our study, all subjects expressed *Clostridium XIV*, while only 13% of the HCR group and 9% of the LCR group expressed *Clostridium IV*, and no significant differences were observed between the HCR and LCR groups. However, *Clostridium* XIV was 2 times higher in the HCR than in the LCR group, which could be in accordance with the study by Li and colleagues [112].

The human oral microbiota has recently become a new focus for its involvement in systemic diseases [113,114]. Our work underlines the presence in the interdental niche of healthy adolescents of a number of oral bacteria described in the literature for their association with systemic diseases. Effectively, *B. dentium* is known to cause bacteraemia [115]. *A. odontolyticus* has been identified in actinomycosis throughout the body [116,117]. *R. dentocariosa* and *S. mutans* are known to be associated with bacteraemia and infective endocarditis [118,119]. *E. corrodens* has been described in cardiovascular diseases (CVD) [120,121], and *C. ochracea* has also been described in lupus [122,123]. *F. nucleatum* has been related to CVD, Alzheimer′ s, and adverse pregnancy [124]. *P. gingivalis* was linked to CVD, respiratory tract infection, cancer, Alzheimer′ s, rheumatoid arthritis, and adverse pregnancy [124,125]. CVD and Alzheimer′ s were also associated with *P. intermedia*, *T. denticola*, and *T. forsythia*, which was also implicated in respiratory tract infection [124]. *C. rectus* and *V. parvula* were linked to CDV [124]. *P. nigrescens* was discovered in rheumatoid arthritis [125]. Therefore, the accumulation of these pathogenic bacteria in the interdental space must be considered. These bacteria represent a risk-predisposing factor to the development of systemic diseases in the future. As interdental hygiene is often neglected (the toothbrush is not used enough), it is necessary to introduce interdental brushing as early as adolescence.

## 5. Conclusions

Estimating the risk of caries associated with bacterial factors in interproximal sites in adolescents will permit a more evidence-based strategy for medical referrals for certain individuals and contribute to a better definition of carious risk status, periodicity and intensity of diagnostic, prevention and restorative services. In our study, the analysis of the interdental microbiota from a sample of adolescents differentiated by level of caries risk highlights the potential role that three bacteria (*S. sobrinus*, *E. corrodens*, and *T. forsythia*) could have on the predictive development of interproximal caries. *E. corrodens* appears to be more interesting because it was found in all interdental sites from the HCR group and, it had the lowest *p*-value when comparing HCR and LCR groups. Moreover, the detection of *S. sobrinus* and *T. forsythia* could give false negatives because they were not detected in all the interdental sites and all the adolescents for the HCR group. While existing tools for caries risk assessment and interproximal adolescence caries prediction have restricted manageable clinical value, introducing a biological, non-subjective and personalized marker could be recommended to increase the CRA classification. Sensitive assessment of metabolic process using microbial biomarkers at the biofilm–enamel interface could thus perform it possible to specify endpoints prior to the clinical expression. The knowledge obtained from our trial contributes to generating active hypotheses related to the composition and variability of the oral microbiome in the interdental space of adolescents. These hypotheses can be prospectively explored in longitudinal cohort studies, using next generation sequencing techniques, essential to identify other biomarkers of the disease in real time.

## Figures and Tables

**Table 1:** Adolescent caries risk assessment.

	High Risk	Low Risk
Biological (interview)		
Patient with low socioeconomic status	Yes	
Patient has >3 between-meal sugar-containing snacks or beverages per day	Yes	
Protective (interview)		
Patient brushes teeth daily with fluoridated toothpaste		Yes
Patient has regular dental care		Yes
Clinical Findings (ICDAS and salivary tests) ICDAS		
Patient had >1 interproximal lesion	Yes	
Patient has active white spot lesions or enamel defects	Yes	
Salivary tests (hydration, salivary consistency, resting saliva pH, stimulated saliva flow, stimulated saliva pH and saliva buffering capacity)		
Patient has high salivary risk	Yes	

**Table 2:** Age, sex, and characteristics of the full mouth of the study group.

	High Caries Risk	Low Caries Risk	*p*-Value
**Subjects**			
Age (years)	16.08 ± 0.81	15.88 ± 0.73	0.49
Gender			0.37
Male	10 (40%)	7 (28%)	
Female	15 (60%)	18 (72%)	
**Full mouth**			
Teeth	27.80 ± 0.82	27.48 ± 1.23	0.89
BOIB (%)	96.55 ± 7.87	96.52 ± 5.13	0.52
PI	1.56 ± 0.51	1.76 ± 0.43	0.22
GI	1.92 ± 0.28	1.72 ± 0.46	0.22
**Interdental space diameter**			0.35
0.8 mm	10 (10%)	10 (10%)	
0.9 mm	22 (22%)	31 (31%)	
1.1 mm	68 (68%)	59 (59%)	

The values are mean ± standard deviation, the numbers and percentages of subjects are indicated. BOIB: Bleeding On Interdental Brushing, GI: Gingival Index, PI: Plaque Index.

**Table 3:** Description (mean ± sd) of bacterial counts (log_10_x + 1) in 200 quadrants (*n* = 50 patients × 4 quadrants/patient) and comparison according to caries risk.

Variable	All (*n* = 200)	Caries Risk		
		High (*n* = 100)	Low (*n* = 100)	*p*-Value
TB	9.42 ± 0.48	9.43 ± 0.47	9.40 ± 0.48	0.796
*B. dentium*	0.76 ± 2.20	0.60 ± 1.95	0.91 ± 2.41	0.522
*Lactobacillus* spp.	4.54 ± 0.67	4.57 ± 0.72	4.50 ± 0.60	0.591
*R. dentocariosa*	5.91 ± 1.14	5.73 ± 1.43	6.08 ± 0.70	0.226
*S. cristatus*	4.41 ± 3.02	4.97 ± 2.83	3.85 ± 3.11	0.096
*S. mutans*	1.96 ± 2.76	1.95 ± 2.81	1.97 ± 2.72	0.969
*S. salivarius*	6.90 ± 0.70	7.05 ± 0.70	6.75 ± 0.67	0.068
*S. sobrinus*	0.30 ± 1.19	0.56 ± 1.58	0.04 ± 0.43	0.049
*S. wiggsiae*	5.85 ± 2.35	6.09 ± 1.88	5.59 ± 2.72	0.412
*A. odontolyticus*	3.77 ± 1.19	3.97 ± 1.18	3.56 ± 1.18	0.120
*V. parvula*	5.05 ± 1.36	5.07 ± 1.38	5.02 ± 1.32	0.881
*A. actinomycetemcomitans*	0.28 ± 1.32	0.55 ± 1.83	0.00 ± 0.00	0.076
*C. ochracea*	4.04 ± 2.26	4.18 ± 2.11	3.89 ± 2.40	0.609
*E. corrodens*	5.84 ± 1.27	6.17 ± 1.01	5.50 ± 1.40	0.006
*S. mitis*	5.35 ± 0.62	5.48 ± 0.63	5.22 ± 0.58	0.075
*S. sanguinis*	7.00 ± 0.98	7.05 ± 1.22	6.94 ± 0.65	0.580
*C. rectus*	5.89 ± 1.79	6.00 ± 1.71	5.77 ± 1.86	0.560
*C. gracilis*	4.22 ± 1.14	4.31 ± 0.89	4.12 ± 1.34	0.524
*F. nucleatum*	7.42 ± 0.54	7.40 ± 0.56	7.43 ± 0.50	0.801
*P. intermedia*	2.85 ± 3.43	3.31 ± 3.53	2.38 ± 3.27	0.153
*P. micra*	5.05 ± 2.50	4.95 ± 2.55	5.14 ± 2.45	0.741
*P. nigrescens*	2.12 ± 1.99	2.43 ± 1.92	1.80 ± 2.01	0.131
*P. gingivalis*	0.34 ± 1.47	0.68 ± 2.02	0.00 ± 0.00	0.082
*T. denticola*	1.82 ± 3.16	2.17 ± 3.39	1.45 ± 2.88	0.345
*T. forsythia*	4.95 ± 3.20	5.66 ± 2.77	4.24 ± 3.43	0.046
*Clostridium IV*	0.43 ± 1.25	0.52 ± 1.39	0.34 ± 1.09	0.441
*Clostridium XIV*	6.39 ± 0.75	6.54 ± 0.67	6.23 ± 0.79	0.078

The colors refer to (i) the colors of the Socransky complexes for the purple, green, yellow, orange, and red colors, (ii) cariogenic bacteria for the pink color and (iii) bacteria from the *clostridium* group for the gray color.

**Table 4:** Distribution of the pathogens according to sites and subjects.

	All				Sex								IDB Size																			
					Male				Female				0.6 mm				0.7 mm				0.8 mm				0.9 mm				1.1 mm			
	Positive Sites ^1^		Positive Subjects ^2^		Positive Sites ^1^		Positive Subjects ^2^		Positive Sites ^1^		Positive Subjects ^2^		Positive Sites ^1^		Positive Subjects ^2^		Positive Sites ^1^		Positive Subjects ^2^		Positive Sites ^1^		Positive Subjects ^2^		Positive Sites ^1^		Positive Subjects ^2^		Positive Sites ^1^		Positive Subjects ^2^	
	HCR	LCR	HCR	LCR	HCR	LCR	HCR	LCR	HCR	LCR	HCR	LCR	HCR	LCR	HCR	LCR	HCR	LCR	HCR	LCR	HCR	LCR	HCR	LCR	HCR	LCR	HCR	LCR	HCR	LCR	HCR	LCR
*n*	100	100	25	25	40	28	10	7	60	72	15	18	0	0	0	0	0	0	0	0	10	10	5	7	22	31	13	20	68	59	21	22
TB	100	100	25	25	40	28	10	7	60	72	15	18	0	0	0	0	0	0	0	0	10	10	5	7	22	31	13	20	68	59	21	22
*Bd*	9	13	6	6	1	2	1	1	8	11	5	5	0	0	0	0	0	0	0	0	2	0	1	0	2	4	2	3	5	9	3	5
*Lspp*	100	100	25	25	40	28	10	7	60	72	15	18	0	0	0	0	0	0	0	0	10	10	5	7	22	31	13	20	68	59	21	22
*Rd*	96	100	24	25	36	28	9	7	60	72	15	18	0	0	0	0	0	0	0	0	10	10	5	7	22	31	13	20	64	59	20	22
*Scri*	77	62	24	22	31	20	10	6	46	42	14	16	0	0	0	0	0	0	0	0	6	3	4	3	17	24	11	15	54	35	21	19
*Smutans*	34	36	15	18	6	10	5	6	28	26	10	12	0	0	0	0	0	0	0	0	5	6	3	4	5	10	5	9	24	20	8	13
*Ssal*	100	100	25	25	40	28	10	7	60	72	15	18	0	0	0	0	0	0	0	0	10	10	5	7	22	31	13	20	68	59	21	22
*Ssob*	12	1	5	1	3	0	1	0	9	1	4	1	0	0	0	0	0	0	0	0	2	0	2	0	4	1	2	1	6	0	3	0
*Sw*	93	82	24	21	34	27	9	7	59	55	15	14	0	0	0	0	0	0	0	0	10	9	5	6	21	25	13	16	62	48	16	18
*Ao*	96	93	25	25	37	27	10	7	59	66	15	18	0	0	0	0	0	0	0	0	9	10	5	7	20	29	12	18	67	54	21	22
*Vp*	95	95	25	25	37	25	10	7	58	70	15	18	0	0	0	0	0	0	0	0	10	10	5	7	22	29	13	19	63	56	21	22
*Aa*	9	0	3	0	3	0	1	0	6	0	2	0	0	0	0	0	0	0	0	0	0	0	0	0	0	0	0	0	9	0	3	0
*Co*	83	76	23	23	27	22	8	6	56	54	15	17	0	0	0	0	0	0	0	0	10	3	5	3	19	28	12	17	54	45	15	19
*Ec*	100	96	25	25	40	27	10	7	60	69	15	18	0	0	0	0	0	0	0	0	10	9	5	6	22	30	13	19	68	57	21	22
*Smitis*	100	100	25	25	40	28	10	7	60	72	15	18	0	0	0	0	0	0	0	0	10	10	5	7	21	31	13	20	68	59	21	22
*Ssan*	98	100	25	25	39	28	10	7	59	72	15	18	0	0	0	0	0	0	0	0	10	10	5	7	21	31	13	20	67	59	21	22
*Cr*	95	93	25	25	37	26	10	7	58	67	15	18	0	0	0	0	0	0	0	0	10	7	5	6	20	30	13	20	65	56	21	22
*Cg*	99	93	25	24	39	27	10	7	60	66	15	17	0	0	0	0	0	0	0	0	10	10	5	7	22	30	13	19	67	53	21	21
*Fn*	100	100	25	25	40	28	10	7	60	72	15	18	0	0	0	0	0	0	0	0	10	10	5	7	22	31	13	20	68	59	21	26
*Pi*	48	36	19	18	17	9	7	4	31	27	12	14	0	0	0	0	0	0	0	0	3	4	2	3	10	9	7	8	35	23	14	13
*Pn*	65	47	21	20	17	10	6	5	48	37	15	15	0	0	0	0	0	0	0	0	5	2	3	2	14	16	9	6	56	29	19	14
*Pm*	81	84	24	24	32	26	10	7	49	58	14	17	0	0	0	0	0	0	0	0	7	8	4	6	17	26	11	15	57	50	20	21
*Pg*	11	0	3	0	4	0	1	0	7	0	2	0	0	0	0	0	0	0	0	0	0	0	0	0	2	0	1	0	9	0	3	0
*Td*	30	21	12	8	10	8	5	4	20	13	7	4	0	0	0	0	0	0	0	0	1	0	1	0	7	6	4	3	22	15	10	6
*Tf*	83	62	24	20	30	14	10	4	53	48	14	16	0	0	0	0	0	0	0	0	9	7	5	5	16	19	10	12	58	36	20	16
*ClosIV*	13	9	7	7	4	5	2	3	9	4	5	4	0	0	0	0	0	0	0	0	0	0	0	0	5	2	5	2	8	7	6	6
*ClosXIV*	100	100	25	25	40	28	10	7	60	72	15	18	0	0	0	0	0	0	0	0	10	10	5	7	22	31	13	20	68	59	21	22

^1^ Positive sites correspond to the number of sites expressing one pathogenic species or the total bacteria (TB). ^2^ Positive subjects indicate the number of subjects expressing one pathogenic species or the total bacteria. The colors refer to (i) the colors of the Socransky complexes for the purple, green, yellow, orange, and red colors, (ii) cariogenic bacteria for the pink color and (iii) bacteria from the *clostridium* group for the gray color. *Aa: Aggregatibacter actinomycetemcomitans, Ao: Actinomyces odontolyticus, Bd: Bifidobacterium dentium, Cg: Campylobacter gracilis, ClosIV: Clostridium cluster IV, ClosXIV: Clostridium cluster XIVa and XIVb, Co: Capnocytophaga ochracea, Cr: Campylobacter rectus, Ec: Eikenella corrodens, Fn: Fusobacterium nucleatum,* HCR: High Carious risk group, LCR: Low Carious Risk group, *Lspp: Lactobacillus spp, n*: total number of sites or subjects tested, *Pg: Porphyromonas gingivalis, Pi: Prevotella intermedia, Pm: Parvimonas micra, Pn: Prevotella nigrescens, Rd: Rothia dentocariosa, Scri: Streptococcus cristatus, Smitis: Streptococcus mitis, Smutans: Streptococcus mutans, Ssal: Streptococcus salivarius, Ssan: Streptococcus sanguinis, Ssob: Streptococcus sobrinus, Sw: Scardovia wiggsiae, Td: Treponema denticola, Tf: Tannerella forsythia, Vp: Veillonella parvula.*

**Table 5:** Interactions (*p*-values) between caries risk and selected variables on bacterial counts (log_10_x + 1) in 200 quadrants (*n* = 50 patients × 4 quadrants/patient).

Variable	*p*-Values Interaction Caries Risk x...		
	Sex	IDB Size	BOIB%
TB	0.835	0.812	0.376
*B. dentium*	0.246	0.009 ^1^	0.208
*Lactobacillus* spp.	0.857	0.332	0.716
*R. dentocariosa*	0.595	0.492	0.348
*S. cristatus*	0.253	0.048 ^1^	0.166
*S. mutans*	0.008 ^1^	0.280	0.057
*S. salivarius*	0.136	0.144	0.023 ^1^
*S. sobrinus*	0.123	0.103	0.147
*S. wiggsiae*	0.139	0.666	0.426
*A. odontolyticus*	0.179	0.331	0.166
*V. parvula*	0.882	0.012 ^1^	0.075
*A. actinomycetemcomitans*	0.203	0.064	0.194
*C. ochracea*	0.124	<0.001 ^1^	0.374
*E. corrodens*	0.020 ^1^	0.070	0.015 ^1^
*S. mitis*	0.299	0.362	0.119
*S. sanguinis*	0.643	0.421	0.034 ^1^
*C. rectus*	0.809	0.199	<0.001 ^1^
*C. gracilis*	0.400	0.752	0.605
*F. nucleatum*	0.853	0.674	0.594
*P. intermedia*	0.421	0.095	0.186
*P. micra*	0.940	0.559	0.105
*P. nigrescens*	0.001 ^1^	0.024 ^1^	0.281
*P. gingivalis*	0.198	0.203	0.209
*T. denticola*	0.762	<0.001 ^1^	0.020 ^1^
*T. forsythia*	0.112	0.130	0.056
*Clostridium IV*	0.306	0.005 ^1^	0.473
*Clostridium XIV*	0.341	0.014 ^1^	0.062

^1^*p* < 0.05. BOIB: Bleeding on Interdental Brushing, IDB: Interdental Brush, TB: Total of bacteria. The colors refer to (i) the colors of the Socransky complexes for the purple, green, yellow, orange, and red colors, (ii) cariogenic bacteria for the pink color and (iii) bacteria from the *clostridium* group for the gray color.

**Table 6:** Stratified analysis of the effect of BOIB on bacterial counts ^1^, according to caries risk level in 200 quadrants (*n* = 50 patients × 4 quadrants/patient).

Variable	Caries Risk	
	High (*n* = 100)	Low (*n* = 100)
*Streptococcus salivarius*	*r* = 0.22, *p* = 0.011	*r* = 0.21, *p* = 0.198
*Eikenella corrodens*	*r* = 0.07, *p* = 0.388	*r* = 0.22, *p* = 0.164
*Streptococcus sanguinis*	*r* = 0.16, *p* = 0.008	*r* = 0.23, *p* = 0.213
*Campylobacter rectus*	*r* = 0.36, *p* < 0.001	*r* = 0.35, *p* = 0.030
*Treponema denticola*	*r* = 0.12, *p* = 0.138	*r* = 0.23, *p* = 0.035

^1^ This stratified analysis is restricted to those situations where the interaction between caries risk and sex (or IDB size) on bacterial counts is significant (*p* < 0.05) (detailed results not shown). The colors refer to (i) the colors of the Socransky complexes for the purple, green, yellow, orange, and red colors, (ii) cariogenic bacteria for the pink color and (iii) bacteria from the *clostridium* group for the gray color.

## Supplementary Materials

The following are available online at https://www.mdpi.com/2076-2607/7/9/319/s1, Table S1: Species-specific and ubiquitous real-time PCR primers for 26 bacteria, annealing temperatures, and the limits of quantification.
