# Supplementary material for: The Oral Bacterial Microbiome of Interdental Surfaces in Adolescents According to Carious Risk
Source: Microorganisms. 2019 Sep 5;7(9):319. doi: 10.3390/microorganisms7090319 (PMC6780098; doi:10.3390/microorganisms7090319)
Supplement: Supplementary file 1 [file microorganisms-07-00319-s001.pdf]

Supplementary Table 1. Species-specific and ubiquitous real-time PCR primers for 26 bacteria, the annealing temperature, and the limit of quantification.

Target	Primer pairs (5'-3')[a]	Reference	Annealing temp (°C)	LOQ (E+02)
TB	CCATGAAGTCGGAATCGCTAGT<br>GCTTGACGGGCGTGTG	Kozarov et al., 2006	66	200
Aa	AAACCCATCTCTGAGTTCTTCTTC<br>ATGCCAACTTGACGTTAAAT	Kobayashi et al. 2008	60	10
Ao	CTTTGGGATAACGCCGGGAAAC<br>CTACCCGTCAAAGCCTTGGT	Suzuki et al., 2007	66	5
Bd	AGCCCATCGCTTAACGGTGGGTC<br>GGCCRTATCTCTACGGCC	Henne et al., 2015	60	680
Cg	AGAGTTTGATCCTGGCTCAG<br>GGACGCATGCCCATCTTTCACCACCGC	Kobayashi et al., 2008	66	5
ClosIV	TTACTGGGTGTAAAGGG<br>TAGAGTGCTCTTGCGTA	Dyke and McCarthy, 2002	60	67
ClosXIV	CGGTACYTGACTAAGAAGC<br>TGGCTACTRDRVAYARGGG	Dyke and McCarthy, 2002	55	70
Co	AGAGTTTGATCCTGGCTCAG<br>GATGCCGCTCCTATATACTATGGGG	Kobayashi et al., 2008	66	5
Cr	TTTCGGAGCGTAAACTCCTTTTC<br>TTTCTGCAAGCAGACACTCTT	Kobayashi et al., 2008	60	20
Ec	GGGAAGAAAAGGGAAGTGCT<br>TCTTCAGGTACCGTCAGCAAAA	Kozarov et al., 2006	60	5
Fn	AGAGTTTGATCCTGGCTCAG<br>GTCATCGTGCACACAGAATTGCTG	Fouad et al., 2002	60	40
Lspp	TGGAAACAGRTGCTAATACCG<br>GTCCATTGTGGAAGATTCCC	Byun et al., 2004	62	10
Pg	AGGCAGCTTGCCATACTGCG<br>ACTGTTAGCAACTACCGATGT	Sakamoto et al., 2001	60	4
Pi	CGTGGACCAAAGATTCATCGGTGGA<br>CCGCTTTACTCCCCAACAAA	Fouad et al., 2002	60	60
Pm	AGAGTTTGATCCTGGCTCAG<br>ATATCATGCGATTCTGTGGTCTC	Fouad et al., 2002	60	60
Pn	ATGAAACAAAGGTTTTCCGGTAAG<br>CCCACGTCTCTGTGGGCTGCGA	Fouad et al., 2002	66	5
Rd	GGGTTGTAAACCTCTGTTAGCATC<br>CGTACCCACTGCAAAACCAG	Tsuzukibashi et al., 2012	66	124
Scri	TCCAATGCCAAACCTTTACT<br>ATACGAGTATCTTCTTCACG	Wang et al., 2009	58	57
Smitis	GAGTCCTGCATCAGCCAAGAG<br>GGATCCACCTTTTCTGCTTGAC	Suzuki et al., 2005	66	5
Smutans	GCCTACAGCTCAGAGATGCTATTCT<br>GCCATACACCACTCATGAATTGA	Yoshia et al., 2003	66	8
Ssal	GTGACGGTAGCTTACCAGAAA<br>CGCTTTACGCCCAATAAATC	Zhou et al., 2016	60	41
Ssan	AGTTGCCATCATTGAGTTG<br>GTACCAGCCATTGTAACAC	Zhou et al., 2016	60	54
Ssob	TGCTCCAGTGTTACTAATGA<br>TAACTCCTCTTATGCGGTATT	Zhou et al., 2016	60	14
Sw	GTGGACTTTATGAATAAGC<br>CTACCGTTAAGCAGTAAG	Henne et al., 2015	51	12
Td	TAATACCGAATGTGCTCATTTACAT<br>CTGCCATATCTCTATGTCATTGCTCTT	Sakamoto et al., 2001	60	10
Tf	GGGTGAGTAACGCGTATGTAACCT<br>ACCCATCCGCAACCAATAAA	Sakamoto et al., 2001	60	80
Vp	GAAGCATTGGAAGCGAAAGTTTCG<br>GTGTAACAAGG-GAGTACGGACC	Igarashi et al., 2009	60	5
